# Physical activity and mortality in a prospective cohort of middle-aged and elderly men – a time perspective

**DOI:** 10.1186/1479-5868-10-94

**Published:** 2013-08-08

**Authors:** Andrea Bellavia, Matteo Bottai, Alicja Wolk, Nicola Orsini

**Affiliations:** 1 Unit of Nutritional Epidemiology, Institute of Environmental Medicine, Karolinska Institutet, Stockholm, Sweden; 2 Unit of Biostatistics, Institute of Environmental Medicine, Karolinska Institutet, Stockholm, Sweden

**Keywords:** Physical activity, Mortality, Survival, Percentiles, Laplace

## Abstract

**Background:**

Higher physical activity (PA) levels are known to be associated with lower risk of death. Less attention, however, has been paid to directly evaluate the effect of PA on the time by which a certain fraction of the population has died.

**Methods:**

A population-based cohort of 29,362 men 45 to 79 years of age was followed from January 1998 to December 2010. A total of 4,570 men died. PA was assessed through a self-administrated questionnaire. Adjusted differences in the number of months by which 10% (10th percentile) of the cohort has died, according to levels of total PA (TPA) and different domains of PA were estimated using Laplace regression.

**Results:**

Overall, the 10th survival percentile was 9.6 years, that is, 90% of the cohort lived longer than 9.6 years. We found a strong evidence of non-linearity between TPA and the 10th survival percentile (P-value < 0.001). Compared to men with the lowest TPA (29 metabolic equivalents (MET)-hrs/day), men with a median TPA (41 MET-hrs/day) had 30 months longer survival (95% CI: 25–35). Below the median TPA, every increment of 4 MET-hrs/day, approximately a 30 minutes brisk pace daily walk, was associated with a longer survival of 11 months (95% CI: 8–15). Above the median TPA additional activity was not significantly associated with better survival.

**Conclusions:**

We found that a physically active lifestyle is associated with a substantial improvement in survival time, up to 2.5 years over 13 years of follow-up.

## Background

The positive effects on health of a physically active lifestyle have been a major objective of public health research in recent years. Regular physical activity (PA) among adults is associated with a decreased risk of all-cause mortality [1-7], and higher mortality has been observed in sedentary people [8-12]. The largest benefit of PA has been observed in moving from sedentary behavior to moderate levels of activity, but even at high levels of activity benefits can accrue from additional PA [13]. The positive impact of PA in reducing the risk of death has been evaluated both according to a score of total PA [3,4,7], and according to different domains of PA [4-6].

Findings from epidemiological studies are mainly presented in terms of relative risks. Less attention, however, has been paid to directly evaluate variation in time (i.e. months, years) by which a certain fraction of the general population has died according to PA levels. Information on survival percentiles can complement the information provided by relative risks and facilitate the interpretation and communication of the results, giving important advantages both in a clinical and in a public health context. A novel approach to estimate survival percentiles, Laplace regression, has been proposed in the biostatistical literature by Bottai and Zhang [14]. An application of this novel regression method for the analysis of a large prospective cohort has been recently illustrated by Orsini et al [15].

Therefore, to examine the role of total PA and the contribution of different domains of PA in predicting time to death, we analyzed survival percentiles in a population-based cohort of middle-aged and elderly men.

## Methods

### Study population

The population-based Cohort of Swedish Men (COSM) was established in 1997–1998, when all eligible men aged 45 to 79 years residing in Västmanland and Örebro counties (central Sweden) received an invitation to participate in the study along with a self-administrated questionnaire. Information was collected on physical activity, body weight and height, smoking habits, alcohol consumption, educational level and other lifestyle factors. A total of 48,850 men returned the questionnaire (49%). This large population-based cohort was representative of Swedish males between 45 and 79 years of age, in terms of age distribution, educational level and prevalence of overweight [16]. This study was approved by the Regional Research Ethics Board at Karolinska Institutet, and all participants gave their informed consent.

In this analysis, we excluded participants who reported incorrect or missing personal numbers (n = 297), those who died before the baseline (n = 55) or reported pre-baseline cancer (n = 2,592), those who had history of CVD (n = 5,069), those who had diabetes (n = 3,204) and those with any missing information about PA (n = 8,271). After exclusions, a total of 29,362 men were included in the analysis.

### Physical activity assessment

Physical activity information was collected by using five questions about usual PA during the previous year. There were six predefined activity levels for work/occupational activity (from mostly sedentary to heavy manual labor) and five to six predefined categories for time spent on different activities: home/household work (from less than 1 h to more than 8 h per day), walking/bicycling (from hardly ever to more than 1.5 h per day), inactive leisure-time (i.e. watching TV/reading, from less than 1 h per day to 6 h per day or more), and exercising (active leisure-time, from less than 1 h to more than 5 h per week). There was also an open question about the number of sleeping hours/day. Based on the compendium of physical activities [17] we assigned to each activity its intensity defined as metabolic equivalents (MET, kcal kg^-1^ h^-1^). Work occupation was assigned a mean MET value from 1.3 (mostly sitting) to 3.9 (heavy manual labour); walking/bicycling - 3.6 MET; home/household work - 2.5 MET; watching TV/reading - 1.2 MET; leisure-time exercise - 5.0 MET; sleep - 0.9 MET. We then multiplied the intensity score of each activity for its reported duration (h) and estimated the total daily activity score (24 h) by adding all specific activities together. Work occupation contributed to 50% of the TPA score. The other four domains of PA and sleep duration contributed about 10% each to the TPA score. The PA questions have been validated using two 7-day activity records that were performed 6 months apart in group of Swedish men 44–78 years of age and were shown to correlate well with total PA (Spearman’s rank correlation between the questionnaire and PA records was 0.56). The same study showed high reproducibility for TPA (correlation between two questionnaires over different seasons was 0.65) [18].

### Case ascertainment and follow-up

From January 1, 1998, through December 31, 2010, during 13 years of follow-up we documented 4,570 deaths. Information on death was ascertained through linkage to the Swedish Register of Death Causes at the National Board of Health and Welfare.

### Statistical analysis

Laplace regression was used to model percentiles of survival time as function of PA while adjusting for potential confounders [14,15].

We performed our main analyses in terms of the 10th percentile of survival time, the point of time by which the first 10% of the cohort has died. Modeling other survival percentiles provided similar results. The measure of exposure-disease association was defined as 10th Percentile Difference (PD), the difference between the 10th survival percentiles in months in two groups of individuals being compared. We first evaluated the 10th PD according to quartiles of Total Physical Activity (TPA).

In the multivariable analyses we adjusted for baseline age (45–49, 50–54, 55–59, 60–64, 65–69, 70–74, and 75–79 years), body mass index (BMI, <25, 25–29, ≥30 kg/m2), alcohol consumption (current <5 g/day, current 5–9 g/day, current 10–19 g/day, current ≥20 g/day, former, never drinker), smoking status and pack-years of smoking (current ≥40, current 20–39, current < 20, former ≥40, former 20–39, former <20, never), and educational level (1–9, 10–12, >12 years). All quantitative potential confounders were included as categorical variables to facilitate estimation of survival percentiles for specific covariates patterns after fitting a multivariable regression model. Modeling potential quantitative confounders as continuous had negligible impact on the association between physical activity and mortality.

In order to assess the dose–response relation between TPA and mortality, we also modeled TPA as a continuous variable by means of restricted cubic splines with three knots (36, 41, and 48 MET-hrs/day). Linearity was evaluated by testing the null hypothesis that the coefficient of the second spline is equal to zero [19]. We graphed the multivariable adjusted differences in the 10th percentile of survival time as function of TPA using the median value of the bottom quartile (36.5 MET-hrs/day) as referent value. To evaluate if there was any age difference in the dose response we replicated this analysis in the strata of younger (<60 years old) and older (> = 60 years old) participants.

We next estimated differences in the 10^th^ percentile of survival according to levels of the different types of daily activities. For each specific activity we dichotomized the participants between active and inactive (walking/ bicycling: active vs ‘hardly ever’; leisure-time exercising: >1 h/week vs <1 h/week; watching TV/reading: <3 h/day vs > =3 h/day; work occupation: active vs ‘mostly sitting’; home/household work: <1 h/day vs > =1 h/day).

We also examined the association according to a fine grid of survival percentiles around the 10th percentile (i.e. 5th-15th). We graphed survival percentiles comparing the group of active participants (top category in all of the different dichotomized activities) and the group of inactive participants (bottom category in all of the different activities), evaluated at the most frequent covariate pattern (Age: 50–54 years, Smoking: ex-smokers with less than 20 pack for years, Alcohol: 10-20 g/day, BMI: 25-29 kg m^-2^, Education: 1–9 years). Setting the covariates at other categories would have no impact on the differences in survival percentiles.

Statistical analyses were performed with Stata, version 12 (StataCorp, TX, USA) [20].

## Results

Baseline characteristics of the study population by quartiles of TPA are shown in Table 1. The median TPA was 41 MET-hrs/day (Interquartile Range = 38-45). Educational level was the only socio-demographic factor unequally distributed across quartiles of TPA. Participants with higher education were more likely to have sedentary jobs and therefore had a lower TPA score; and vice versa. Distributions of age, BMI, smoking, and drinking status were similar across quartiles of TPA.

**Table 1:** **Age**-**standardized baseline characteristics by quartiles of total physical activity in 45 to 79**-**year**-**old Swedish men**

	**Quartiles of total physical activity, range (median), MET**^**a**^**-hrs/day**			
Characteristics^b^	<38 (36.5)	38-41 (39.5)	41-45 (43)	>45 (47.5)
No. of subjects	7,354	7,329	7,339	7,340
Mean age at baseline, year	58.1	58.4	59.8	58.9
Mean body mass index, kg m^-2^	25.9	25.5	25.5	25.4
Smoking status, %				
Current	25	22	24	24
Former	38	39	38	37
Never	37	39	38	39
Drinking status, %				
Current	93	93	92	91
Former	3	2	3	3
Never	4	5	5	6
Education (yrs), %				
1-9	53	57	72	83
10-12	21	19	13	8
Greater than 12	26	24	15	9

^a^MET = metabolic equivalent.

^b^All factors except age were directly standardized to the age distribution of the entire study cohort (n = 29,362).

In 13 years of follow-up we documented 4,570 deaths distributed between CVD (30%), cancer (30%), and other causes (40%). The distribution of causes of death was similar across levels of TPA (Pearson’s Chi-squared test P-value = 0.63). Overall, the 10th survival percentile was 9.6 years, that is, 90% of the cohort lived longer than 9.6 years.

We first assessed the 10th survival PD according to quartiles of TPA (Table 2). Comparing with men in the bottom quartile (median 36.5 MET-hrs/day) and adjusting for baseline age, men in the top quartile (median 47.5 MET-hrs/day) lived 14 months longer (95% CI: 10–19). The association between TPA and survival time was slightly attenuated after adjustment for BMI, alcohol consumption, smoking status, and educational level. Compared with men in the bottom quartile, men in the top quartile of TPA lived 13 months longer (95% CI: 7–18). We performed a sensitivity analysis to address the issue of potential reverse causation due to undiagnosed diseases (all known chronic diseases were excluded at baseline). After exclusion of participants who died during the first 3 years of follow-up we observed that the multivariable adjusted association (top vs. bottom quartile of TPA) was 10 months (95% CI: 6–14).

**Table 2:** Multivariable adjusted 10th survival percentile differences (PD) in months by quartiles of total physical activity

	**Total physical activity, range (median), MET**^**a**^**-hrs/day**				
		<38 (36.5)	38-41 (39.5)	41-45 (43)	>45 (47.5)
Model 1 (Age)	PD (95% CI), months	Ref	13 (9–18)	11 (7–15)	14 (10–19)
Model 2 (Further)^b^	PD (95% CI), months	Ref	11 (6–16)	9 (5–14)	13 (7–18)

^a^MET = metabolic equivalent.

^b^10th survival PD, differences in time (months) by which ten percent of the cohort has died, were adjusted for baseline age (45–49, 50–54, 55–59, 60–64, 65–69, 70–74, and 75–79 years), body mass index (BMI, <25, 25–29, ≥30 kgm2), alcohol consumption (current <5 g/day, current 5–9 g/day, current 10–19 g/day, current ≥20 g/day, former, never drinker), smoking status and pack-years of smoking (current ≥40, current 20–39, current < 20, former ≥40, former 20–39, former <20, never), and educational level (1–9, 10–12, >12 years).

**Figure 1:**
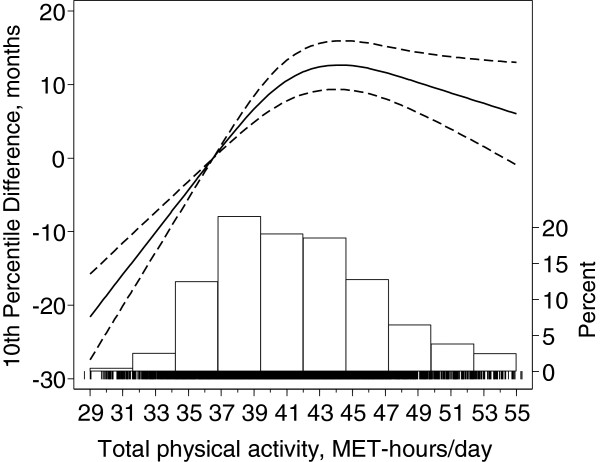
**Tenth survival percentile differences, differences in months by which ten percent of the cohort has died as function of total physical activity.** Data were fitted using a Laplace regression model with restricted cubic splines with 3 knots (36, 41 and 48 MET-hrs/day) of the distribution of total physical activity. The estimates were adjusted for baseline age, body mass index, alcohol consumption, smoking status, and educational level. Dashed lines represent 95% confidence limits. The reference value of total physical activity is the median of the bottom quartile (36.5 MET-hrs/day). Tick marks represent position of men who died. The histogram is the distribution of total physical activity in the cohort.

We next investigated the dose–response relation between TPA and time to death (Figure 1). We found a strong evidence of non-linearity (P-value < 0.001) between TPA and the time interval by which 10% of the cohort die. Compared to men with the lowest TPA (29 MET-hrs/day), men with a median TPA (41 MET-hrs/day) had 30 months longer survival (95% CI: 25–35). Below the median TPA, every increment of 4 MET-hrs/day, approximately a 30 minutes brisk pace daily walk, was associated with a longer survival of 11 months (95% CI: 8–15). Above the median TPA additional activity was not significantly associated with better survival. Extreme PA levels tended to be associated with shorter survival time, although survival percentile differences between extremely high values and the median value of TPA were not statistically significant (Figure 1).

**Figure 2:**
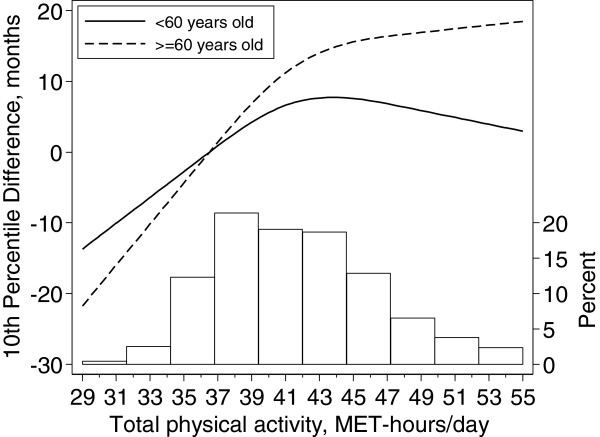
**Tenth survival percentile differences as function of total physical activity among younger (<60) and older (> = 60) participants.** Data were fitted using a Laplace regression model with restricted cubic splines with 3 knots (36, 41 and 48 MET-hrs/day) of the distribution of total physical activity. The estimates were adjusted for baseline age, body mass index, alcohol consumption, smoking status, and educational level. The reference value of total physical activity is the median of the bottom quartile (36.5 MET-hrs/day).

Figure 2 shows the age-stratified dose–response association between TPA and the 10th percentile of survival. Older men seem to accrue more benefits from regular PA. The difference in survival between those with the lowest level of TPA (29 MET-hrs/day) and the median TPA (41 MET-hrs/day) was 20 months (95% CI: 9–31) for men under 60 years of age and 33 months (96% CI: 20–46) for the over 60. However, the shape of the dose–response was similar between older and younger participants.

Table 3 provides mutually adjusted associations of different domains of PA and the 10th survival percentile. Compared with men who hardly walked or biked, those men who engaged in this leisure time activity lived 11 months longer. Men who spent more than 1 h/week in exercising lived 8 months longer than men who spent less than 1 h/week. Inactivity during leisure time (sitting watching TV or reading) limited to 3 h/day was associated with 8 months improved survival compared to sitting more than 3 h/day. Men with an active work occupation lived 6 months longer than men who mostly sat at work.

**Table 3:** Multivariable adjusted 10th survival percentile differences (PD) in months by different domains of physical activity

**Specific physical activity**	**PD**^**a **^**(95% CI), months**
Walking/Bicycling (Active vs hardly ever)	11 (7–16)
Exercise (>1 h/week vs <1 h/week)	8 (4–12)
Limited inactivity -watching TV/reading- (<3 h/day vs > =3 h/day)	8 (4–12)
Work occupation (Active vs mostly sitting)	6 (4–10)
Home/household work (>1 h/day vs <1 h/day)	−2 (−6-2)

^a^ The different domains of physical activity, were mutually adjusted using the inactive group as reference. Furthermore, estimates were adjusted for baseline age (45–49, 50–54, 55–59, 60–64, 65–69, 70–74, and 75–79 years), body mass index (BMI, <25, 25–29, ≥30 kgm2), alcohol consumption (current <5 g/day, current 5–9 g/day, current 10–19 g/day, current ≥20 g/day, former, never drinker), smoking status and pack-years of smoking (current ≥40, current 20–39, current < 20, former ≥40, former 20–39, former <20, never), and educational level (1–9, 10–12, >12 years).

**Figure 3:**
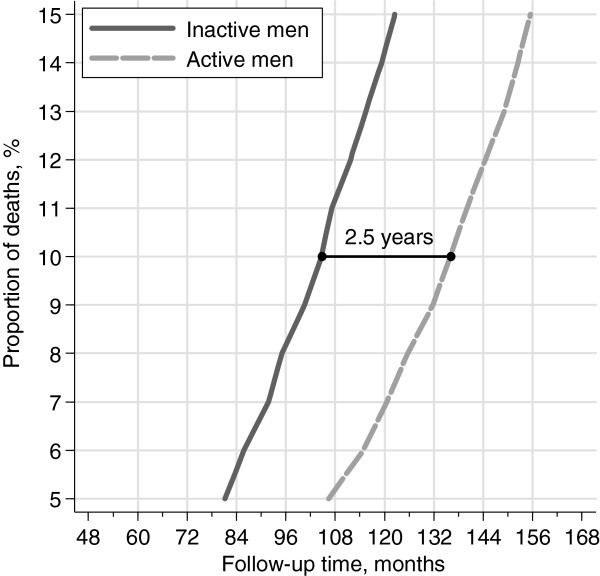
**Survival percentiles (proportion of the cohort who has died) among the group of inactive participants (solid line, walking/bicycling-hardly ever, exercise- < 1 h/week, tv watching/reading > 3 h/day, work occupation-mostly sitting) and the group of participants active in different activities (dashed line, else) estimated with multivariable Laplace regression.** The estimates were adjusted for baseline age, body mass index, alcohol consumption, smoking status and educational level; in the graph these covariates are fixed on the most frequent category (Age: 50–54 years, Smoking: ex-smokers with less than 20 pack for week, Alcohol: 10-20 g/day, BMI: 25-29 kg m^-2^, Education: 1–9 years).

We next compared the group of inactive participants (hardly ever walk or bike, exercise less than 1 hours per week, sitting watching TV/reading 3 hours per day or more, mostly sitting at work, and less than 1 hour per day of home/household work) and the group of participants who were active in the different kinds of daily activity (engaged in leisure time walking/bicycling, exercise more than 1 hours per week, limited leisure time inactivity - watching TV/reading - to less than 3 hours per day, physically active at work, and more than 1 hour per day of home/household work) (Figure 3). Active men lived 31 months longer than those men who were inactive (95% CI: 23–39). The difference in survival was not limited to the 10th percentile but was substantial throughout the 13 years of follow-up, ranging from 25 months for the 5th percentile (95% CI: 15–34) to 33 months for the 15th percentile (95% CI: 25–40).

## Discussion

We found that increasing TPA was strongly associated with longer survival time. During 13 years of follow-up, men with the median level of TPA lived 2.5 years longer than those men with the lowest TPA. In the subpopulation of men below the median TPA we observed an improvement of approximately 11 months every increment of 4 MET-hrs per day - the equivalent of a 30 minutes brisk pace daily walk. Above the median TPA increasing activity was not associated with better survival.

Physical activity was examined according to a score of TPA (MET-hrs/day) and according to different domains of PA. In recent years the MET approach has become the standard approach to code physical activity from self-reported questionnaire [17]. The score of TPA provides a continuous measure of physical activity and allows modeling the dose–response relationship with flexible approaches. However, this score is obtained by adding the value of different domains of physical activity and is therefore sensitive to the number of administered questions. To facilitate the interpretation of our results we also evaluated the specific types of physical activity (e.g. minutes of walking/bicycling, hours of leisure-time exercise, hours of inactivity), which can make easier conveying our findings into accessible public messages. In our cohort, participants with the lowest total physical activity were more likely to be in the lowest category of each domain (walking/biking hardly ever, leisure time exercise less than 1 h/week, watch TV/read more than 3 h/day, working mostly sitting, and house-work less than 1 h/day). Participants with a median total physical activity, which was associated with the best survival, were more likely to be men with 20–40 minutes per day of walking or biking, 2-3 h/week of exercising, 1-2 h/day of watching TV or reading, work occupation sitting half of the time, and more than 1 h/day of house-work. This mix of physical activities appears to be in agreement with the current recommendation of physical activity for older adults (e.g. 30–60 min of moderate-intensity activity a day, muscle-strengthening activities at least twice a week, reduce sedentary behaviors) [21,22]. Our findings support the recommended level of physical activity and suggest that every decrement of 4 MET-hrs/day (i.e. reducing daily walking/biking by 30 minutes, reducing weekly exercising by 45 min., increasing daily watching TV/reading by 1.5 hrs) from this optimal level, is progressively associated with shorter survival time.

An important strength of this study is that we presented results directly in terms of survival time. Many epidemiological studies examined the association between PA and all-cause mortality and presented the findings in terms of relative risks. A recent systematic review and meta-analysis of cohort studies about PA and all-cause mortality by Woodcock et al. [13] concluded that being physically active reduces the risk of all-cause mortality and that the largest benefits are obtained when moving from no activity to low levels of activity (summary relative risk was 0.81). When results are reported only in terms of relative risk (i.e. 19% risk reduction) it is not clear, however, what is the background risk for the referent population or how this background risk varied over the follow-up period. In addition, the lack of time dimension in the epidemiological measure of association may constitute a major limitation to the general public media, to which research findings should ultimately be addressed.

The advantage of a percentile-based approach in comparing survival experiences is that it combines information on risk and time. The estimation of survival percentiles allows evaluating the magnitude of the association in terms of the unit of time of the event of interest, providing a reasonable estimate of the probability of experiencing the event after a given time point. This information has a direct, intuitive interpretation; for instance, if the 10th percentile of time to death is 10 years, there is a 10% probability that a randomly selected individual from the population will die within 10 years. Therefore, the estimation of survival percentiles can potentially simplify the communication of the results and may encourage people to make healthy changes to their lifestyle. To the best of our knowledge, this is the first large-population based study that provides adjusted survival percentiles differences in subpopulations defined by PA levels.

Survival percentiles can be derived from the survival function estimated by the well-known Kaplan-Meier method. This non-parametric method, however, does not allow modeling the effects of continuous exposures, adjusting for confounders or assessing interactions in predicting survival. Laplace regression overcomes these limitations providing a complementary tool for analysis of observational studies. The percentile-based approach is also different from accelerated failure-time models, a commonly used parametric method to model survival time [23]. Accelerated failure-time models, which assume a log-linear relationship between the time to event and a set of covariates, do not model percentiles of survival time directly. Regression estimates indicate the direction of the acceleration factors (i.e. acceleration/deceleration). In order to obtain information on time to the event of interest post-estimation calculations, often not straightforward, are required. Laplace regression models survival percentiles, and its coefficients can be directly interpreted as differences in survival percentiles (i.e. days, months, years) without requiring any transformation of the parameters. Moreover, Laplace regression allows modeling one or more survival percentiles simultaneously and testing variation of exposure effects across survival percentiles [14,15].

Besides the use of a novel percentile-based approach for the analysis of prospective observational study, other strengths of our study include the large size of the cohort, its population-based and prospective design, the large number of cases, and the completeness of case ascertainment through the National Register. These features can increase the generalizability of the study findings. The study population included middle-aged and elderly men, and our findings may not be generalizable to women and young men.

A limitation of this study is that measures of PA were self-reported. A certain degree of exposure misclassification is therefore inevitable. However, in prospective studies any misclassification would be non-differential and would most likely attenuate rather than exaggerate any observed relationships. Our validation study, a self-administrated structured 7-day PA diary, indicated relatively good reproducibility and validity of self-reported PA [18]. We acknowledge, however, the lack of an objective measure of PA (e.g. accelerometers).

In summary, in a cohort of middle-aged and elderly men, we found that a physically active lifestyle is associated with a substantial improvement in survival time, up to 2.5 years over 13 years of follow-up.

## Competing interests

All authors declare that they have no competing interests.

## Authors’ contributions

AB and NO formulated the original research ideas in collaboration with AW. AB performed the analyses and drafted the manuscript in collaboration with NO. MB provided a critical methodological contribution regarding the statistical approach. AW oversaw the research and revised the draft. All authors contributed to, read and approved the final version of the manuscript.
